# Extraparenchymal neurocysticercosis: Demographic, clinicoradiological, and inflammatory features

**DOI:** 10.1371/journal.pntd.0005646

**Published:** 2017-06-09

**Authors:** Mariana Marcin Sierra, Mariana Arroyo, May Cadena Torres, Nancy Ramírez Cruz, Fernando García Hernández, Diana Taboada, Ángeles Galicia Martínez, Tzipe Govezensky, Edda Sciutto, Andrea Toledo, Agnès Fleury

**Affiliations:** 1 Research Unit on Neuroinflammation, Instituto de Investigaciones Biomédicas - Universidad Nacional Autónoma de México / Facultad de Medicina – Universidad Nacional Autónoma de México / Instituto Nacional de Neurología y Neurocirugía, México City, México; 2 Immunology Department, Instituto de Investigaciones Biomédicas – Universidad Nacional Autónoma de México, México City, México; 3 Neurocysticercosis Clinic, Instituto Nacional de Neurología y Neurocirugía, México City, México; Ludwig-Maximilians-University, UNITED STATES

## Abstract

**Background:**

Extraparenchymal neurocysticercosis (ExPNCC), an infection caused by *Taenia solium* cysticerci that mainly occurs in the ventricular compartment (Ve) or the basal subarachnoid space (SAb), is more severe but less frequent and much less studied than parenchymal neurocysticercosis (ParNCC). Demographic, clinical, radiological, and lumbar cerebrospinal fluid features of patients affected by ExPNCC are herein described and compared with those of ParNCC patients.

**Methodology and principal findings:**

429 patients with a confirmed diagnosis of neurocysticercosis, attending the Instituto Nacional de Neurología y Neurocirugía, a tertiary reference center in Mexico City, from 2000 through 2014, were included. Demographic information, signs and symptoms, radiological patterns, and lumbar cerebrospinal fluid (CSF) laboratory values were retrieved from medical records for all patients. Data were statistically analyzed to assess potential differences depending on cyst location and to determine the effects of age and sex on the disease presentation. In total, 238 ExPNCC and 191 ParNCC patients were included. With respect to parenchymal cysts, extraparenchymal parasites were diagnosed at an older age (*P* = 0.002), chiefly caused intracranial hypertension (*P* < 0.0001), were more frequently multiple and vesicular (*P* < 0.0001), and CSF from these patients showed higher protein concentration and cell count (*P* < 0.0001). SAb patients were diagnosed at an older age than Ve patients, and showed more frequently seizures, vesicular cysticerci, and higher CSF cellularity. Gender and age modulated some traits of the disease.

**Conclusions:**

This study evidenced clear clinical, radiological, and inflammatory differences between ExPNCC and ParNCC, and between SAb and Ve patients, and demonstrated that parasite location determines different pathological entities.

## Author summary

Neurocysticercosis (NCC) is caused by the establishment of *Taenia solium* larvae in the human central nervous system. While NCC diagnosis, treatment, and prevention have clearly improved in the last 40 years, the disease still causes significant morbidity and mortality in endemic regions of Latin America, Asia, and Africa. Herein we described demographic, clinical, radiological, and cerebrospinal fluid features of a large series of NCC patients, and evaluated the relevance of parasite location on disease presentation. 191 patients with parenchymal parasites, 125 patients with extraparenchymal cysts, and 113 patients lodging parasites in both locations were included. Our results clearly demonstrated that differences in parasite location actually determines distinct diseases, with wide variations in severity. This is particularly evident when comparing parenchymal with extraparenchymal patients and ventricular with subarachnoid patients. Gender and age also modulate some characteristics of the disease. In conclusion, parasite location is one of the most important features of the disease, which must be considered when approaching an NCC patient.

## Introduction

Neurocysticercosis (NCC) is caused by the establishment of the larval stage of *Taenia solium* in the central nervous system. NCC is still endemic in a significant portion of low-income countries in Asia, Africa, and Latin America. The recent but highly noticeable emergence of human NCC in the USA and to a lesser extent in some European countries has fostered a growing medical concern about this neglected parasitic disease [1, 2].

One of the most evident traits of NCC is a great clinical heterogeneity [3, 4]. Several elements are relevant in this observation, like patient-related factors (age, sex, genetic background) and disease-related issues (number, stage, and location of parasites) [5, 6]. With respect to parasite location in the central nervous system, most cysts are located in the parenchyma or in the sulci of the subarachnoid space of the convexity (ParNCC). While these parasites are actually lodged in two different compartments, they are generally grouped together as they share common characteristics in term of symptoms, diagnosis, treatment, and prognosis, and because sometimes it is difficult to differentiate them by neuroradiological studies. ParNCC has been extensively studied: Its main symptom is epilepsy [7, 8]; the parasite can be easily recognized by neuroradiological studies [9], and cysticidal treatment clearly improves most patients [10,11]. On the contrary, much less is known about extraparenchymal NCC (ExPNCC), occurring when cysticerci are lodged in the subarachnoid space of the basal cisterns, Sylvian fissure, and spinal medulla or in the ventricular system; such lack of information is probably due to a lower prevalence and the higher difficulties for its diagnosis [12, 13]. Although reviews and case reports of ExPNCC have been published [14–22], large series of patients are scarce. Some of them were conducted before MRI was extensively used [23, 24], and thus fail to describe it thoroughly. Other papers are mainly focused on treatment [25, 26], and the most recent one, describing all NCC cases diagnosed in one hospital at the United States between 1997 and 2005, only reports 35 ExPNCC cases [27].

The appearance of parasites in this location, especially in the subarachnoid space, is frequently different from ParNCC. They generally appear like a cluster of grapes, and thus were termed racemose cysts. They occur when cysts are lodged in a place that allows for unusual growth, and arise from the segmentation of cysticercus cellulosae following the development of interconnected new cysts, which generally lack of a scolex [28]. They are larger than parenchymal cysts and are identified on magnetic resonance imaging (MRI) as multiple cystic lesions [29].

Considering this context, the aim of this study was to describe the demographic, clinical, radiological, and lumbar CSF features of ExPNCC patients and to compare them with ParNCC cases.

## Methods

This is a descriptive study including patients with a definitive diagnosis of neurocysticercosis attending the Instituto Nacional de Neurología y Neurocirugía (INNN), in Mexico City. INNN is a tertiary referral center with the most advanced diagnosis tools in Mexico. Due to this, INNN attracts severe patients, particularly those with ExP parasite location. While it serves patients lacking social security from all the Mexican territory, most attending patients are from Central Mexico.

### Standard protocol approvals

This study was conducted in accordance with local clinical research regulations and was approved by the Institutional Review Board and Ethical Committee of the Instituto Nacional de Neurología y Neurocirugía (Mexico City). All patients (parents or guardians in the case of children) gave written informed consent, and all analyzed data were anonymized.

### Patients

In total, 429 Mexican patients who attended for the first time the INNN Neurocysticercosis Clinic in Mexico City from 2000 through 2014 were included in this study. Patients attending the Clinic before 2007 were retrospectively included by retrieving information from each patient’s clinical and radiological records. From 2007 onwards, patients were included prospectively. NCC diagnosis was based on pathology results (50 patients, 11.6%), imaging findings (only cranial computed tomography in 16 patients, all of them with calcified disease only, showing no symptoms suggesting extraparenchymal locations, and both computed tomography and magnetic resonance imaging in all others), clinical and lumbar CSF features, as well as the response to specific treatment. Response to specific treatment was defined as the occurrence of any change in size (decrease) or intensity (increase) in one or more parasites, 4 to 6 months after treatment. All patients included were regarded as ParNCC or ExPNCC, considering the latest diagnosis criteria [30].

**Fig 1:**
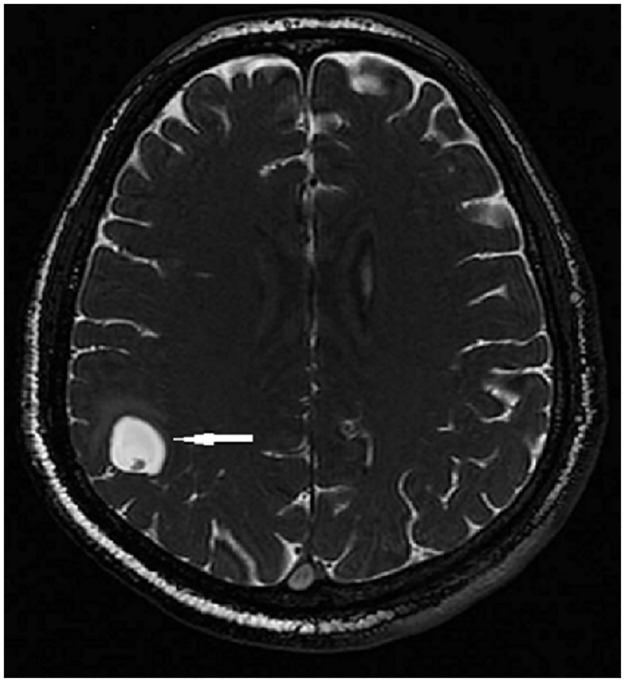
Single parenchymal cysticerci in right parietal lobe (MRI Fast Imaging Employing Steady-state Acquisition, FIESTA).

**Fig 2:**
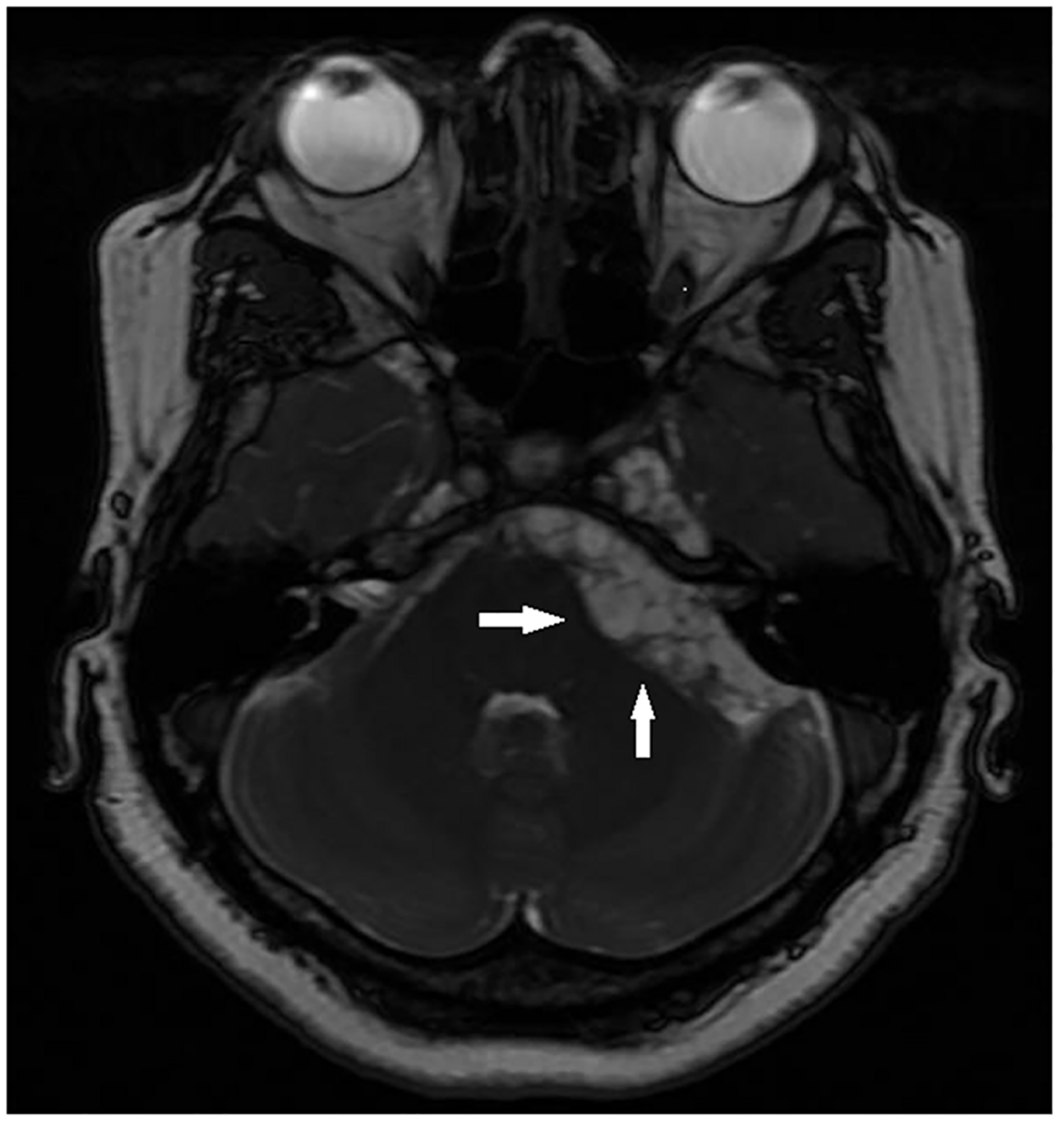
Multiple cysticerci located in the left pontocerebellar cistern (MRI FIESTA).

**Fig 3:**
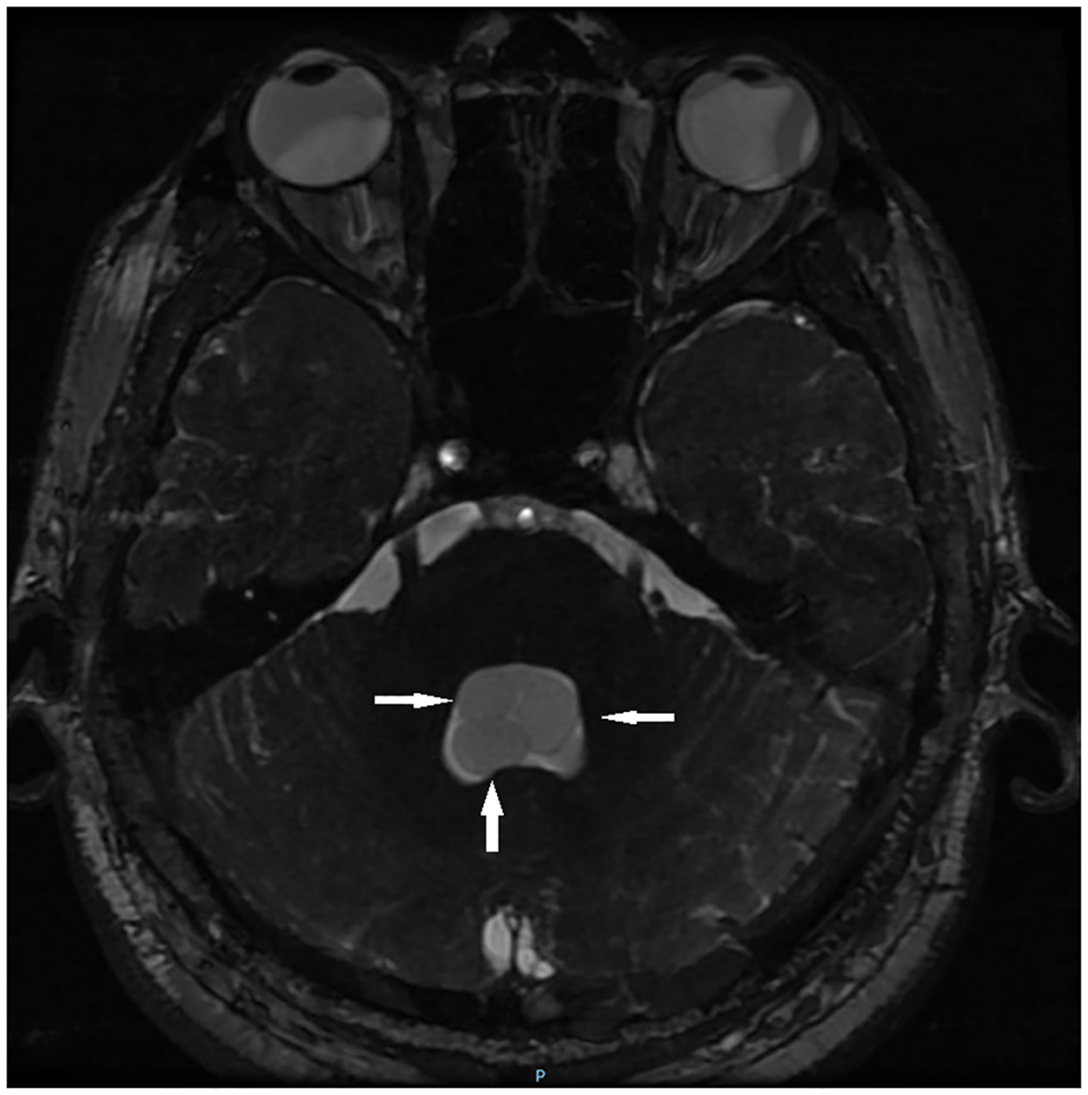
Example of multiple cysticerci located in the fourth ventricle (MRI FIESTA).

Patients were divided into three groups depending on parasite location, according to imaging studies: Par (parenchyma or subarachnoid sulci of the convexity), ExP (subarachnoid space of the Sylvian fissure, basal cisterns, medulla, and ventricles), and mixed (Mx, parenchymal and extraparenchymal). Examples of parenchymal, subarachnoid, and ventricular parasites are shown in Figs 1, 2 and 3, respectively.

Parasite degenerating stages were defined according to the classical scale, as published elsewhere [30]. Racemose parasites were defined as multiple vesicular parasites without scolex located in one same extraparenchymal region.

All patients included were diagnosed in the INNN and had not received specific NCC treatment before. All patient features herein reported (clinical, radiological, and inflammatory) are pre-treatment.

The assessed variables were: age at diagnosis, sex, area of provenance, symptoms, lumbar CSF characteristics, and the number and degenerative stage of cysts. The presence of specific antibodies and of the HP10 antigen in lumbar CSF and serum was assayed by ELISA in a subset of patients, using previously published protocols [31, 32].

### Statistical analysis

Data were processed in Excel (Microsoft, Redmond, VA) and SPSS 15.0 (SPSS Inc., Chicago, IL), and reported as proportion or mean ± standard deviation. Statistical comparisons between variables were performed using either parametrical or non-parametrical tests, depending on data distribution. Categorical variables were compared by the chi-squared test with Yates correction or Fisher’s exact test, while mean comparison was made using Student’s *t*-test, ANOVA (with Bonferroni or Tamhane post-hoc test when appropriate), or Mann-Whitney test. Correlations between numeric variables were assessed using the Pearson coefficient of correlation and checking for outliers and leverage. Normality was checked using the Kolmogorov-Smirnov test. Dichotomous dependent variables were analyzed using logistic regression to take into account the possible effects of confounding variables. For the same purpose, continuous dependent variables were analyzed using multifactorial analysis of variance, including covariables. Variance homogeneity was tested using Levene’s test; Welch correction was used when variances were not statistically similar. Differences were regarded as statistically significant when P-value was less than 0.05.

## Results

### Main features of included patients

Of the total 429 patients included, 125 showed extraparenchymally-located parasites (ExP), 191 showed parenchymally-located parasites (Par), and 113 showed cysts in both compartments (ExP+Par: Mx). Most patients (359, 83.7%) were from urban areas, defined as having a population of more than 2500 inhabitants (National Institute of Statistics, INEGI, Mexico).

**Fig 4:**
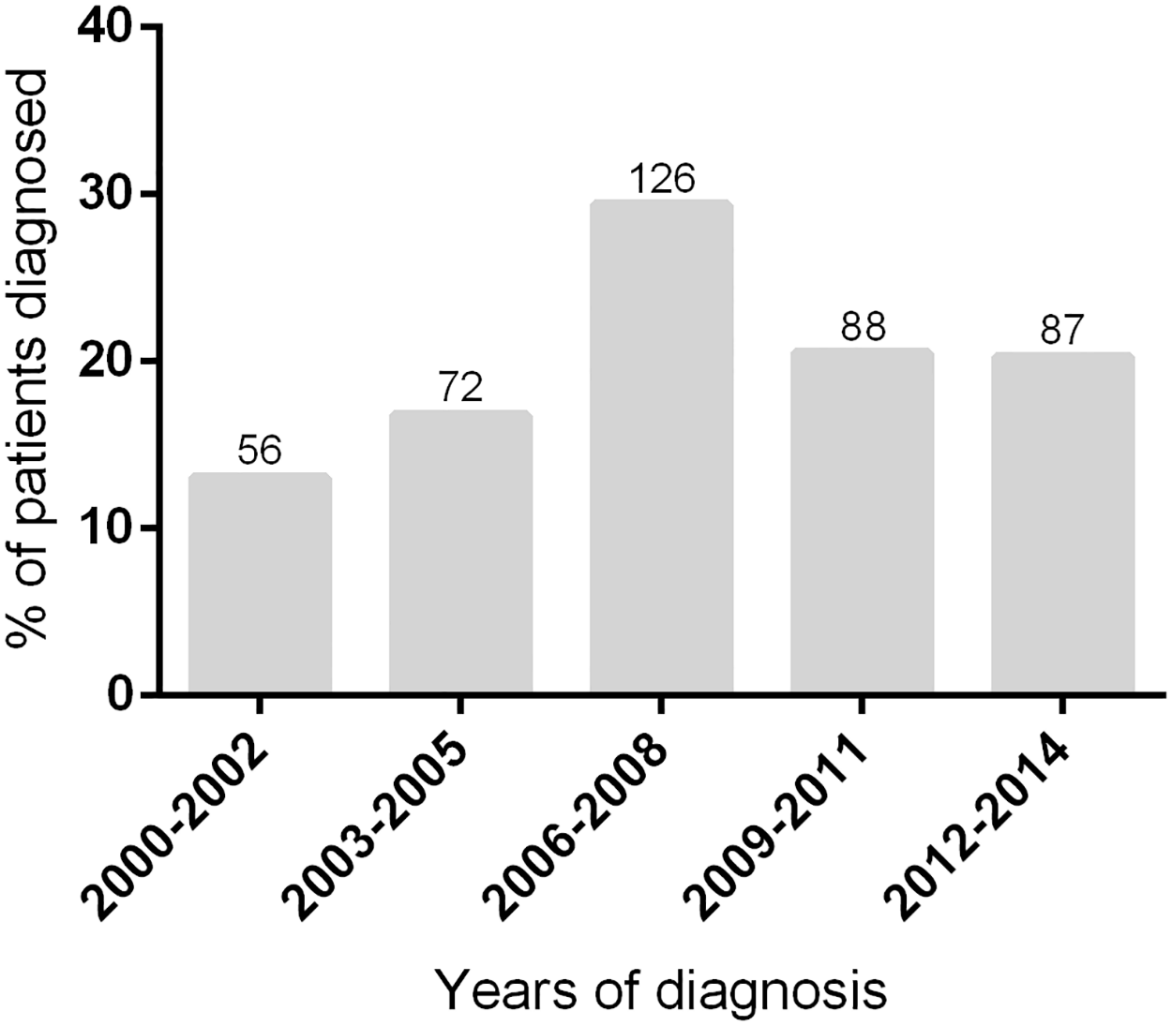
Year of diagnosis of included patients. Numbers at the top of each column represent the number of patients diagnosed in the assessed period.

The number of patients diagnosed by year varied from 14 (in 2000) to 46 (in 2008), with no significant trend in the period of inclusion (*R* = 0.46, *P* = 0.44) (Fig 4).

The main characteristics of the patients included in the three groups are shown in Table 1.

**Table 1:** Demographic, clinical, radiological, and inflammatory features of the three patient groups.

	Extraparenchymal neurocysticercosis *N* = 125	Extraparenchymal and parenchymal *N* = 113	Parenchymal cysticercosis *N* = 191	*P*_1_	*P*_2_
**Age at diagnosis**	41.3 ± 14.2†	42.2 ± 11.4*	37.8 ± 12.8*†	0.007	0.002
**Sex (Male)**	70 (56.0)	54 (47.8)	88 (46.1)	0.21	0.21
**Rural**	13 (10.4)†	27 (23.9)†	30 (15.7)	0.018	0.76
**Symptoms**					
Headache	34 (27.2)	34 (30.1)	53 (27.7)	0.87	0.85
Seizures	28 (22.4)†	32 (28.6)*	147 (77.0)*†	<0.0001	<0.0001
ICH	90 (72.0)*¥	64 (56.6)†¥	12 (6.3)*†	<0.0001	<0.0001
Focal deficit	23 (18.4)*	12 (10.6)	19 (9.9)*	0.07	0.14
Psychiatric	3 (2.4)	5 (4.4)	6 (3.1)	0.67	0.9
**Lumbar CSF characteristics**					
Cells (/mL)	96.7 ± 163.2†	103.6 ± 351.2*	13.8 ± 48.4*†	0.003	0.001
Protein concentration (mg/dL)	219.4 ± 691.4†	126.4 ± 190.2*	42.1 ± 45.9*†	0.006	0.006
Glycorrhachia (mg/dL)	45.1 ± 26.1	47.7 ± 25.4	60.8 ± 26.2	<0.0001	<0.0001
**Antibodies**	90 (94.7)†	85 (94.4)*	41 (44.6)*†	<0.0001	<0.0001
**Antigens**	43 (76.8)†	43 (76.8)*	9 (45)*†	0.01	0.004
**Degenerating stage of parasites**^¥^					
Vesicular	113 (90.4)†	101 (89.4)*	68 (35.6)*†	<0.0001	<0.0001
Single	22 (19.5)†	14 (13.9)*	38 (55.9)*†	<0.0001	<0.0001
Degenerating	24 (19.2)†*	35 (31.0)*	58 (30.4)†	0.05	0.19
Single	9 (37.5)†	16 (45.7)*	43 (74.1)*†	0.002	<0.0001
Calcification	0*¥	100 (88.5)†¥	123 (64.4)*†	<0.0001	<0.0001

Categorical variables are reported as *N* (%). Continuous variables are reported as mean ± standard deviation. *P*_1_: comparing the three groups of patients. *P*_2_: comparing extraparenchymal (with or without parenchymal cysts) and parenchymal forms. Similar symbols in the same line in two patient groups indicate significant differences between them. ICH: Intracranial hypertension. Characteristics of lumbar CSF were available in 117 Par patients (60.9%), 112 ExP (89.6%), and 104 (92.8%) Mx patients. The presence of antibodies was assayed in 95 ExP, 89 Mx and 93 Par patients; the presence of antigens was assayed in 56 ExP, 56 Mx, and 20 Par patients.

^¥^ Since patients could lodge parasites in different stages, the total sum of parasites in each stage is higher than the number of patients (please see S1 Table for a more precise description on this respect).

#### Parenchymal NCC

As previously reported, seizures were the main symptom in parenchymal NCC. Most patients showed generalized seizures (63.8%), while the others (37.2%) presented partial seizure with or without secondary generalization. Intracranial hypertension was present in only 6.3% of the patients and it was associated with an increase in CSF cellularity and protein concentration (*P* = 0.02 and *P* = 0.002, respectively), but not with the number of parasites (*P* = 0.12). Seizures were associated with the presence of degenerating parasites (*P* = 0.005).

While patients lodging parasites in these locations usually showed normal lumbar CSF, 8 of them (6.8%) had CSF cellularity counts higher than 50/mL, and 6 of them (5.1%) showed protein concentrations higher than 100 mg/dL. Specific antigens in serum and CSF and specific antibodies in CSF were present in less than one half of patients (S2 Table).

Most parasites were calcified, and similar proportions were in vesicular and degenerating stage.

#### Extraparenchymal NCC and mixed (extraparenchymal + parenchymal) NCC

The demographic, clinical, radiological, and inflammatory characteristics of ExPNCC patients with or without parenchymal cysts were similar (Table 1).

Subarachnoid cisterns were the most frequent location in ExP and Mx patients (78 [62.4%] vs. 69 [61.1%], *P* = 0.83), followed by the ventricular system (50 [40%] vs. 46 [40.7%], *P* = 0.91), Sylvian fissure (24 [19.2%] vs. 18 [15.9%], *P* = 0.51), and medullar space (6 [4.8%] vs. 2 [1.8%], *P* = 0.29). The only differences were a lower ICH incidence (72.0% vs. 56.6%, *P* = 0.01) and a higher proportion of degenerating cysts (31.0% vs. 19.2%, *P* = 0.05) in Mx with respect to ExP patients. The difference in the proportion of degenerating cyst between these groups could be due to the presence of parenchymal degenerating cyst in the Mx group. Indeed (S1 Table), the proportion of patients in the Mx group lodging degenerating parasites in the extraparenchymal compartment was 27/113 (23.9%), not significantly different from the proportion of degenerating parasites in ExP patients (19.2%, *P* = 0.43).

In view of the high similarities between these two groups, ExPNCC and Mx patients (ExpMx = 238) were categorized together for further analysis.

The precise location of parasites in patients from the ExpMx group is shown in Table 2.

**Table 2:** Location of extraparenchymal cysts.

Basal Subarachnoid cysts	
Single location	95
Associated with medullar	3
Associated with Sylvian	16
Ventricular parasites	
Single location	62
Associated with Sylvian	1
Basal subarachnoid + ventricular cysts	
Single location	32
Associated with Sylvian	1
Sylvian	
Single location	23
Associated with medullar	1
Medullar	
Single location	4
TOTAL	238

As shown, most patients (184, 77.3%) lodged cysticerci in a single ExP location, mostly in the basal subarachnoid cisterns (95, 51.6%), followed by the ventricular system (62, 33.7%), Sylvian fissure (23, 12.5%), and subarachnoid space of medulla (4, 2.2%). With respect to multiple location (54 patients), more than half (32, 59.2%) presented both cysticerci in the basal subarachnoid cisterns and in the ventricles.

Most parasites in the ventricular system were located in the fourth ventricle (55, 57.3%), followed by the lateral (16, 16.7%) and third ventricle (13, 13.5%). The other 12 patients exhibited multiple cysts in the different ventricles.

Sixteen patients (38%) presented cysts over 2.5 cm in diameter in the Sylvian fissure.

In the ExP group, 91 of the 113 vesicular parasites (80.5%) were racemose. Parasites were mainly located in the subarachnoid space (70, 76.9%), but also in the ventricular system (11, 12.1%), the Sylvian fissure (9, 9.9%), and the spinal subarachnoid space (1, 1.1%).

Symptoms related to intracranial hypertension were the most frequently observed (154, 65%); they were associated with an increase in CSF protein concentration (*P* = 0.02). No patient suffered vascular events at the initial presentation, but 28 of 102 (27.4%) had asymptomatic lacunar infarcts as shown on MRI. Patients with lacunar infarcts (50.1 ± 12.1) were significantly older than patients without this sign (40.1 ± 11.5, *P* < 0.0001). When comparing patients with and without asymptomatic lacunar infarcts, the first group showed higher total cholesterol (253.7 ± 74.2 vs. 195.8 ± 31.1 mg/dL, *P* = 0.04) and triglyceride levels (257.8 ± 109.8 vs. 179.1 ±111.4 mg/dl, *P* = 0.04), while glycemic levels were not significantly different (*P* = 0.55). Parasite location was similar in both groups, with most parasites located in the subarachnoid space (71.4% vs. 68.9%, *P* = 0.81).

A high variability was observed in CSF inflammatory features. Mean cellularity was close to 100/mL, but 52 patients (24%) exhibited a cell count less than or equal to 15/mL, and 20 patients (9.2%) showed counts over 200/mL. Most cells (91%) were lymphocytes. A similar variability was observed in protein levels: 60 patients showed protein concentration less than 40 mg/dL, and 26 patients exhibited concentrations higher than 300 mg/dL. CSF specific antigen was detected significantly more frequently in vesicular parasites (81.4%) than in degenerating ones (57.1%), while detection of specific antibodies was above 85% in both vesicular and degenerating cysts (S2 Table).

In univariate analysis, antigen detection was significantly associated with parasite multiplicity (OR: 4.67, CI95%: 1.9–11.4, *P* = 0.001), with the presence of vesicular parasites (OR: 4.58, CI95%: 1.8–11.49, *P* = 0.001), and with parasite extraparenchymal location (OR: 5.23, CI95%: 2.15–12.73, *P* < 0.0001). In multivariate analysis, none of these associations remained statistically significant.

### Differences between ExpMx and ParNCC patients

Table 1 (*P*_2_) shows that ExPMx patients are diagnosed at an older age than Par patients, and also exhibited intracranial hypertension, inflammatory CSF and racemose cysts in a significantly higher frequency.

Similar results were obtained in a multivariate analysis, controlling for gender and age. ICH (OR: 27.1, CI95%: 14.2–51.9, *P* < 0.0001), the presence of antibodies and antigens (OR: 20.6, CI95%: 9.56–44.3, *P* < 0.0001, and OR: 6.2, CI95%: 2.15–17.9, *P* = 0.001, respectively), higher CSF cellularity and protein concentration (*P* < 0.0001), and the presence of vesicular cysts, racemose cysts, and multiple degenerating cysts (OR: 15.86, CI95%: 9.38–26.8, *P* < 0.0001; OR: 6.0, CI95%: 3.3–10.9, *P* < 0.0001; OR: 5.00, CI95%: 2.0–10.4, *P* < 0.0001, respectively) were more frequently observed in ExPMx than in Par patients, while seizures were associated with the Par group (OR:10.39, CI95%: 6.5–16.6, *P* < 0.001).

### Effect of gender and age on clinical, radiological, and CSF characteristics of extraparenchymal cases

Male ExPMx patients were diagnosed at an older age than female patients (43.5 ± 13.0 vs. 39.9 ± 12.6, *P* = 0.03); degenerating cysticerci were more frequent in females than in males (36, 31.6% vs. 22, 17.9%, *P* = 0.01); CSF cellularity was higher in females (105.3 ± 359.4 vs. 95.7 ± 149.2, *P* = 0.05), and CSF protein concentration was higher in males (236.9 ± 677.2 vs. 108.6 ± 230.3, *P* = 0.001).

Vesicular cysticerci were observed at an older age than other parasite stages (42.2 ± 12.6 years vs. 37.5 ± 15.2 years), although the difference was at the limit of statistical significance (*P* = 0.06). Additionally, protein concentration increased with age (*P* < 0.05), and this result persisted when controlling for gender (*R* = 0.14; *P* = 0.04). Neither gender nor age modulated the clinical presentation of ExPMx patients.

### Effect of gender and age on clinical, radiological, and CSF characteristics of parenchymal cases

Regarding clinical features, males showed higher seizure frequency (*P* = 0.004), while females showed a higher ICH frequency (*P* = 0.03); headache was associated with an older age (41.4 ± 12.6 vs. 36.3 ± 12.7, *P* = 0.01), while seizures correlated with a younger age (42.2 ± 12.0 vs. 36.4 ± 12.8 years, *P* = 0.009). Multivariate analysis only confirmed that seizures were associated with male gender (OR: 3.39; CI95%: 1.6–7.3). With regard to parasite degenerative stage, uni- and multivariate analysis showed that vesicular parasites were more frequent in males (OR: 2.23; CI95%: 1.2–4.1), while patients lodging only calcified parasites at diagnosis time were more frequently female (OR: 2.0; CI95%:1.1–3.6). No other differences between genders or associated with age were observed, particularly with respect to CSF characteristics.

### Differences in demographic, symptomatology, imaging, and CSF characteristics between subarachnoid and ventricular parasites

Two main groups of ExPMxNCC patients can be distinguished: those lodging cysticerci in the ventricles (Ve) and those with parasites in the basal subarachnoid cisterns (SAb). Comparing these two groups by univariate analysis after excluding those patients showing parasites in both compartments (Table 3), age at diagnosis was significantly earlier in Ve patients. Seizures, CSF inflammatory parameters, multiple vesicular parasites, and the presence of specific antibody and antigens were more frequent in SAb patients, while the presence of single degenerating parasites was more frequent in Ve patients. In multivariate analysis controlling for age and gender, the association of the following variables with Sab location persisted: seizures (OR: 4.33, CI95%: 1.63–11.52, *P* = 0.003), CSF cellularity (*P* = 0.002) and CSF protein concentration (*P* = 0.008), antibody presence (OR: 7.39, CI95%: 1.28–42.64, *P* = 0.025), and antigen presence (OR: 8.04, CI95%: 2.57–25.14, *P* < 0.0001), presence of vesicular cysts (OR:18.44, CI95%; 3.95–85.99, *P* < 0.0001), and presence of racemose cysts (OR: 25.89, CI95%: 8.3–80.2, *P* < 0.0001).

**Table 3:** Trait differences between subarachnoid and ventricular neurocysticercosis.

	Subarachnoid neurocysticercosis *N* = 114	Ventricular neurocysticercosis *N* = 63	*P*
**Age at diagnosis**	44.2 ± 11.8	36.2 ± 12.0	< 0.0001
**Sex (male)**	62 (54.4)	29 (46.0)	0.29
**Symptoms**			
Headache	34 (29.8)	13 (20.6)	0.18
Seizures	31 (27.2)	8 (12.7)	0.03
ICH	73 (64.0)	46 (73.0)	0.22
Focal deficit	20 (17.5)	6 (9.5)	0.15
Psychiatric	3 (2.6)	0 (0.0)	0.55
**Asymptomatic lacunar infarcts**	17 (27.0)	7 (36.8)	0.40
**Lumbar CSF characteristics**			
Cells (/mL)	116.3 ± 187.9	49.1 ± 59.8	0.001
Protein concentration (mg/dL)	166.6 ± 238.6	86.6 ± 114.1	0.005
Glycorrhachia (mg/dL)	42.5 ± 25.3	56.2 ± 24.6	0.001
**Antibodies**	88 (97.8)	41 (85.4)	0.009
**Antigens**	55 (85.9)	14 (50.0)	< 0.0001
**Degenerating stage of parasites**			
Vesicular only	91 (79.8)	41 (65.1)	0.04
Single	6 (6.6)	22 (53.6)	< 0.0001
Degenerating only	2 (1.7)	17 (27.0)	< 0.0001
Single	0 (0)	10 (58.8)	0.21
Vesicular + degenerating	21 (18.4)	5 (7.9)	0.08
**Association with Parenchymal calcification**	49 (43.0)	29 (46.0)	0.70

Differences in clinical features, cyst degenerating stage, and CSF inflammatory characteristics were evaluated in patients with positive and negative antigen detection. While no differences in the evaluated variables were found in both groups, a tendency (*P* = 0.07) to higher CSF protein concentration was noted in HP10-negative SAb patients.

## Discussion

The demographic, clinical, radiological, and inflammatory status at diagnosis time of 238 ExPNCC was herein compared with that of 191 ParNCC patients. This study represents the first extended patient series with confirmed ExPNCC, the less frequent but also the most severe NCC form.

With respect to demographic variables, male/female ratio was not statistically different between ParNCC and ExPNCC patients, nor between SAb and Ve patients. On the other hand, patients with ExPNCC were diagnosed at a significantly older age than patients with parenchymal cysts; among ExPNCC patients, age at diagnosis was significantly older in patients with subarachnoid than ventricular parasites. In this respect, it is interesting to note that age at diagnosis of Par patients was similar to Ve patients. This may be due to the fact that subarachnoid parasites must reach larger sizes than parenchymal and ventricular ones before causing symptoms. Indeed, parasites in the subarachnoid compartment have no contact with cerebral structures when their size is small due to the wide space available in the subarachnoid cisterns. Symptoms will only be evident when cysts reach such a size that they obstruct CSF flow or have a mass effect affecting cerebral structures; additionally, when cysts have contact with arachnoid/ependymal tissues or blood vessels, they cause signs related with arachnoiditis/ependymitis or vasculitis. So, latency period between infection and symptoms appearance is probably much longer in patients with subarachnoid parasites than in other locations.

Most patients came from urban areas, as reported in previous studies [33]. Some considerations should be made to correctly interpret this finding. First, we defined rurality as proposed by the Mexican Institute of Statistic and Geography (INEGI), official organism that regards communities of less than 2500 inhabitants as rural. We are conscious that, in the context of this study, such definition could be improved. Particularly, it is very likely that in some communities classified as “urban” (having more than 2500 inhabitants), conditions that favor the parasite life cycle are still prevalent. Another point to consider is, as we have commented before, the time between infection and diagnosis, which can be very long (years). While progress has not reached all Mexican territory, a progressive improvement in the socioeconomic status in some regions has allowed us to prevent the completion of *T*. *solium* life cycle in those settings. It is possible that some patients were infected years before, when their living area was more rural than today. Finally, the role of migration from rural to urban areas could be involved as well. Currently, most Mexicans (77.8%) live in urban settings [34] in contrast with the situation in the 1960s, where about one-half of the population (50.7%) lived in this condition. It is possible that patients were infected while living in rural areas but were diagnosed later, after migrating to an urban center. The significantly higher proportion of rural patients in the mixed (parenchymal and extraparenchymal) group is probably a consequence of a higher infectious pressure and the ensuing higher infectious burden, resulting in cyst establishment in different compartments of the central nervous system.

With respect to clinical presentation, our results confirmed that parasite location (ExP vs. Par) in fact determine two entirely different diseases. Intracranial hypertension is the most frequent symptom in ExPNCC patients, affecting more than 64% of them. In contrast, only a small minority (6.3%) of Par patients exhibited this symptom. The association of ICH with higher CSF protein concentration but not with parasite multiplicity in the Par group points to the probable participation of associated arachnoiditis (related with the previous presence of cysts in the SAb compartment, already degenerated at the diagnosis time) rather than of encephalitis in its onset. On the other hand, seizures were the most frequent symptom in ParNCC patients (77%), while only 25.6% of ExP cases showed this condition. Interestingly, although clinical features of subarachnoid and ventricular forms of the disease were similar, seizures were significantly more frequent when parasites were located in the subarachnoid space (*P* = 0.04). This result was not related with the higher frequency of parenchymal parasites in SAb patients, since Par parasites were present in 52.4% and 49.1% of Ve and SAb parasites, respectively (*P* = 0.75). The reason of these differences is not known, but it is feasible that the higher CSF inflammatory response observed in SAb patients may favor vasculitis, resulting in parenchymal vascular insults and triggering seizures [35]. With respect to this point, it is interesting to note that some authors reported an increase in the frequency of hippocampal sclerosis in ParNCC, and it is likely that inflammation-mediated damage of hippocampal neurons is involved in this finding [36].

The distribution of parasite degenerating stages showed some dependence on location. Parasites were mostly vesicular in ExPNCC patients, while the most frequent stage in ParNCC cases was calcification. Similarly, while vesicular cysts were the most frequent degenerating stage in SAb and IV locations (ExPNCC cases), the proportion of vesicular parasites was significantly higher in SAb than in IV parasites. Interestingly, the higher frequency of vesicular parasites was associated with more severe CSF inflammatory parameters. This association was clear in ExP parasites (compared with Par ones) and in subarachnoid parasites (compared with ventricular ones). So, it seems that the local inflammatory reaction was not associated with parasite death in extraparenchymal locations. This scenario is very different from the case of Par parasites, where the local inflammatory reaction is clearly associated with parasite degeneration. In these cases, a close contact between the parasite, brain immune cells and blood vessels, and the disruption of the blood-brain-barrier allowing the arrival of peripheral immune cells, participate in the success of the immune response [37]. Another factor that could be involved in the varying effectivity of the immune response depending on the compartment where it occurs is the presence of regulatory cells in CSF [38]. These cells could play a role in regulating the immune response, decreasing its effectivity in this compartment and favoring parasite survival. The apparent higher effectivity of the immune response in the ventricular compartment with respect to the subarachnoid space could be related with the more restricted space available for a ventricular parasite, which rapidly contacts ependymal cells. Indeed, an experimental study using the related parasite *Mesocestoides corti* showed that ependymal cells actively express immune mediators in experimental NCC, and that ependyma is a prominent source of leukocyte infiltration into the ventricles [39].

While sex and age did not modulate the clinical presentation of ExPMxNCC patients, the only significant result of multivariate analysis in ParNCC cases was a higher frequency of seizures in male patients. Similar results were found in other studies [40, 41], but the explanation is not clear. The influence of these demographic variables on radiological and inflammatory features of NCC was more evident. Indeed, vesicular parasites were significantly more frequent in male Par patients, whereas calcified cysts were the most frequent presentation in female patients. In ExPMx patients, females showed more degenerating parasites and a higher CSF cellularity, while CSF protein concentration was higher in males and in older patients. Altogether, these findings point to a higher intensity and immediacy of the inflammatory reaction in females, as reflected by the increase in CSF cellularity and by a higher frequency of degenerating and calcified parasites. In males, on the other side, the increase in protein concentration probably indicates a more chronic evolution; this possibility is supported by the positive correlation between CSF protein concentration and age. These findings are probably related with immune-endocrine interactions not completely understood yet [4, 42, 43].

Finally, it is relevant to mention that although parasite location (parenchymal or extraparenchymal) determine two clearly different clinical diseases, there is a significant heterogeneity in the intensity of the associated inflammatory reaction and the radiological characteristics within each group. This is particularly evident in the ExP group. CSF was normal in some patients, while an intense inflammatory reaction was observed in others. At a radiological level, although most parasites were racemose, about 20% of patients presented a single parasite. The consequences of this variability are not completely understood, but it could be involved in the heterogeneity of the response to treatment and in the varying success of such treatment, as previous studies indicate [44, 45]. Further analysis is required to better understand this aspect.

In conclusion, this study strongly supports the relevance of parasite location to define the pathology associated to NCC. Therefore, cyst location should be one of the main factors to be considered when approaching a NCC patient.

## Supporting information

S1 Table Relation between localization and stages of the parasites. Par: Parenchymal parasites / ExPar: Extraparenchymal parasites.
(DOCX)

S2 Table Antibodies and antigen detection in the included patients. Similar symbols in the same column in two patient groups indicate significant differences between them.
(DOCX)

S1 Checklist STROBE checklist. (DOC)
