# Supplementary material for: Extraparenchymal neurocysticercosis: Demographic, clinicoradiological, and inflammatory features
Source: PLoS Negl Trop Dis. 2017 Jun 9;11(6):e0005646. doi: 10.1371/journal.pntd.0005646 (PMC5479594; doi:10.1371/journal.pntd.0005646)
Supplement: S1 Table — Par: Parenchymal parasites / ExPar: Extraparenchymal parasites. (DOCX) [file pntd.0005646.s001.docx]

**S1 Table. Relation between localization and stages of the parasites**

|  | **Extraparenchymal neurocysticercosis**  ***N* = 125** | **Extraparenchymal and Parenchymal**  ***N* = 113** | **Parenchymal cysticercosis**  ***N* = 191** |
| --- | --- | --- | --- |
| Vesicular only | 101 | 5 | 32 |
| Degenerating only | 12 | 2 | 28 |
| Calcification only | --- | ---- | 82 |
| Vesicular + degenerating | 12 | 6  Vesicle:   - 1 only in Par - 5 Par + ExPar   Degenerating:   - 1 Par only - 1 ExPar only - 4 Par + ExPar | 8 |
| Vesicular + calcification | --- | 73  Vesicle:   - 59 ExPar only - 14 Par + ExPar   Calcification: all Par | 19 |
| Degenerating + calcification | --- | 10  Degenerating:   - 8 ExPar only - 2 Par + ExPar   Calcification: all Par | 13 |
| Vesicular + degenerating + calcification | --- | 17  Vesicle:   - 12 ExPar - 5 Par + ExPar   Degenerating:   - 7 Par - 9 ExPar - 1 Par + ExPar   Calcification: all Par | 9 |

Par: Parenchymal parasites / ExPar: Extraparenchymal parasites
