# Supplementary material for: Extraparenchymal neurocysticercosis: Demographic, clinicoradiological, and inflammatory features
Source: PLoS Negl Trop Dis. 2017 Jun 9;11(6):e0005646. doi: 10.1371/journal.pntd.0005646 (PMC5479594; doi:10.1371/journal.pntd.0005646)
Supplement: S2 Table — Similar symbols in the same column in two patient groups indicate significant differences between them. (DOCX) [file pntd.0005646.s002.docx]

**S2 Table. Antibodies and antigen detection in the included patients**

|  | Antigen detection | | Antibodies detection |
| --- | --- | --- | --- |
|  | Sera | CSF | CSF |
| Vesicular parasites |  |  |  |
| Total | 59/96 (61.4%)* | 83/110 (75.4%)* | 179/210 (85.2%)* |
| Extra-parenchymal | 56/84 (66.7%) | 79/97 (81.4%)† | 161/169 (95.3%) |
| Parenchymal | 3/12 (25.0%) | 4/13 (30.8%) | 18/41 (43.9%) |
| Only degenerating parasites |  |  |  |
| Total | 6/13 (46.1%)* | 6/12 (50.0%)* | 23/38 (60.5%)* |
| Extra-parenchymal | 4/8 (50%) | 4/7 (57.1%)† | 12/14 (85.7%) |
| Parenchymal | 2/5 (40.0%) | 2/5 (40.0%) | 11/24 (45.8%) |

Similar symbols in the same column in two patient groups indicate significant differences between them.
